# Inhibition of insulin fibrillation by osmolytes: Mechanistic Insights

**DOI:** 10.1038/srep17599

**Published:** 2015-11-30

**Authors:** Sinjan Choudhary, Nand Kishore, Ramakrishna V. Hosur

**Affiliations:** 1 UM-DAE Centre for Excellence in Basic Sciences, Mumbai University Campus, Mumbai 400098, India; 2 Department of Chemistry, Indian Institute of Technology-Bombay, Mumbai 400076, India; 3 Department of Chemical Sciences, Tata Institute of Fundamental Research, Homi Bhabha Road, Mumbai 400005, India

## Abstract

We have studied here using a number of biophysical tools the effects of osmolytes, betaine, citrulline, proline and sorbitol which differ significantly in terms of their physical characteristics such as, charge distribution, polarity, H-bonding abilities etc, on the fibrillation of insulin. Among these, betaine, citrulline, and proline are very effective in decreasing the extent of fibrillation. Proline also causes a substantial delay in the onset of fibrillation in the concentration range (50–250 mM) whereas such an effect is seen for citrulline only at 250 mM, and in case of betaine this effect is not seen at all in the whole concentration range. The enthalpies of interaction at various stages of fibrillation process have suggested that the preferential exclusion of the osmolyte and its polar interaction with the protein are important in inhibition. The results indicate that the osmolytes are most effective when added prior to the elongation stage of fibrillation. These observations have significant biological implications, since insulin fibrillation is known to cause injection amyloidosis and our data may help in designing lead drug molecules and development of potential therapeutic strategies.

Deposition of amyloid fibrils in tissues is associated with amyloidogenic disorders such as Alzheimer’s, Parkinson’s, mad cow diseases, cystic fibrosis, diabetes type II, etc^1^,^2^. This is intimately connected with proteins getting partially unfolded under some stress^3^. In view of such important observations and disease implications considerable effort has been devoted to understand the fibrillation process of proteins and then to screen the compounds that have ability to interfere with the same^4^,^5^,^6^,^7^.

Osmolytes are small organic molecules, several of which have been found to protect proteins from denaturation, misfolding, aggregation and amyloid formation under stressed environmental conditions^8^,^9^,^10^,^11^. Sugars, methylamines, polyols and some amino acids fall into this category and are found to reduce denaturation and aggregation of many proteins^12^,^13^,^14^,^15^,^16^,^17^,^18^,^19^. Osmolytes such as trehalose, ectoine and betaine have been found to protect enzyme functioning in extreme hot and cold environments^20^,^21^,^22^. Another osmolyte, citrulline, present in wild watermelon leaves has been found to be associated with high drought tolerance of the plant^23^. Some other molecules like ectoine, hydroxyectoine, mannosylglyceramide, and mannosylglycerate were found to be very effective against PrP106–126 and Aβ-peptide aggregation^24^,^25^.

Insulin is a small protein hormone of 51 residues and plays a crucial role in glucose uptake. In muscle and adipose tissues it binds to specific transmembrane receptors and stimulates cell signalling pathway which leads to the transport of glucose across the cell membrane^26^. Insulin predominantly contains α-helical structure in its native form and exists as a mixture of monomeric, dimeric and hexameric states depending upon the solvent conditions^27^,^28^,^29^. In the form of hexamers, insulin is packed in the pancreatic storage vesicles and is associated with the efficient conversion of proinsulin to insulin along with its protection from chemical degradation^30^. Several human diseases such as insulinoma, polycystic ovary syndrome, metabolic syndrome and Type I and Type II diabetes^31^,^32^ are associated with improper functioning of insulin production, specifically, insufficient production and aggregation/fibrillation. In the former case insulin has to be supplemented from external sources and in these situations, proper storage of insulin to prevent aggregation/fibrillation^33^,^34^ becomes crucial. Due to the large social impact of insulin related diseases, insulin-based pharmacological formulation has attracted interest of many researchers in the last decades. In this context^33^,^34^,^35^, *in vitro* study of chemical and physical stability of insulin and factors that cause aggregation/fibrillation^36^,^37^,^38^,^39^,^40^ is of great medical importance and interest for developing improved insulin delivery systems^41^,^42^,^43^,^44^.

There have been several reports in the literature on inhibition of fibrillation of proteins, in general^4^,^5^,^6^,^7^,^14^,^15^,^16^,^17^,^18^,^19^, and these include the use of different osmolytes^12^,^13^,^14^,^15^,^16^,^17^,^18^,^19^. Several disaccharides such as maltose, sucrose and trehalose have also been reported to increase the nucleation period of insulin fibrillation at a concentration of ~300 mM^45^. Recently, the inhibitory effects of different quinones on insulin fibrillation have also been reported^46^. However, an understanding of the mechanism of the inhibition process by these agents in terms of intermolecular interactions and associated energies, which is crucial for developing effective drugs, is still very much lacking. In this background, the current work is focussed on searching for effective osmolytes which would inhibit insulin fibrillation, on one hand, and on understanding the mechanism of the same, on the other. For this purpose we have chosen the osmolytes betaine, citrulline, proline and sorbitol (Fig. 1), which have different physical characteristics with regard to polarity, charge distribution, H-bonding abilities etc. A combination of spectroscopic, microscopic and calorimetric techniques has been used to derive structural and thermodynamic information. Isothermal titration calorimetry results provided valuable insights into the mechanism of inhibition of fibrillation and indicated contributions from direct interaction between insulin and the osmolytes. These findings will be useful in providing leads to synthetic chemists to design suitable inhibitors which can act against amyloid formation at low concentrations.

## Results and Discussion

### Fibril formation by insulin

Fibril formation by different proteins is commonly studied using Thioflavin T (ThT) fluorescence assay. ThT is a cationic benzothiazole dye which interacts mainly with amyloid fibrils and gives characteristic emission maxima at 480 nm when excited at 450 nm^47^. The onset and saturation of ThT signal are typically related to the lag time and fibril amount respectively. Although this is valid in most cases, some deviations may arise and then it is necessary to use additional evidences. Microscopic and spectroscopic techniques provide useful additional supports.

Figure 2A shows the time course of fibrillation of insulin as seen by ThT fluorescence assay when incubated at pH 2.0 and 37 °C with stirring at 250 rpm. The formation of insulin fibrils was also confirmed by transmission electron microscopy and scanning electron microscopy which showed clear branched insulin fibrils when the images were taken after 600 min of incubation (Fig. 2C,D). It is seen from Fig. 2A that fibrillation follows a characteristic sigmoidal curve consisting of an initial lag phase, a subsequent elongation phase and a final saturation phase. The fitting of equation 1 (see Materials and methods) to the data thus obtained gives time periods of lag phase as (406 ± 5) min, elongation phase as 406–500 min, and saturation phase thereafter. The apparent rate constant (k_app_) for the growth of fibrils is estimated as 0.04 min^−1^.

The changes in secondary structure of the protein during the course of fibrillation were analyzed by using far-UV CD spectra. The far UV CD spectra of the native protein (before incubation) and after incubation at 37 °C at different time intervals are shown in Fig. 2B. The native insulin shows significant α-helical structure which is characterized by the presence of two minima at 208 nm and 222 nm^48^. As the fibrillation proceeds, the ellipticity at these two wavelengths decreases progressively up to 300 min after which a single minimum starts appearing at ~220 nm (Fig. 2B) which is characteristic of the presence of β-sheets^48^,^49^. Put together, the fluorescence, circular dichroism and microscopic observations establish that insulin forms amyloid fibrils under the studied experimental conditions.

### Effect of osmolytes on insulin fibrillation

We studied the effects of osmolytes betaine, citrulline, proline and sorbitol on insulin fibrillation. These osmolytes have varying characteristics with regard to polarity, charge, H-bonding abilities etc, all of which can lead to different kinds of interactions with the protein surface. Betaine is highly charged, whereas sorbitol has many -OH groups which can participate in many H-bonds with the protein. Proline has a closed ring structure in its side chain which can provide a hydrophobic face. To explore the best conditions for the inhibition process, the experiments were carried out with osmolyte concentrations of 50 mM, 100 mM and 250 mM. For kinetic studies 3 mg ml^–1^ insulin solution was incubated at 37 °C and pH 2.0 under stirring conditions at 250 rpm in presence of different concentrations of osmolytes. The process of fibrillation was monitored by using amyloid specific dye ThT.

Figure 3A represents the kinetic studies of insulin fibrillation in the absence and presence of different concentrations of betaine. The ThT fluorescence kinetics plot indicates that the lag period of insulin fibrillation has slightly decreased at 50, 100 and 250 mM concentrations to (322 ± 3) min, (319 ± 8) min and (360 ± 5) min, respectively, under the studied conditions. However, there is a significant reduction in the ThT fluorescence intensity. It is clear from the figure that betaine has successfully suppressed fibril formation although it has not delayed the onset of fibrillation, wherever it is seen. In the case of citrulline, the lag periods of insulin fibrillation at concentrations of 50 and 100 mM, are found to be (335 ± 4) and (351 ± 3) minutes which becomes (547 ± 6) minutes when the concentration of the osmolyte is raised to 250 mM (Fig. 3B). There is significant decrease in the ThT fluorescence intensity which suggests that the amount of fibrils formed is substantially decreased in the presence of citrulline. The lag periods are (413 ± 3), (472 ± 5) and (569 ± 5) min in the presence of 50, 100 and 250 mM proline, respectively. Here, the fluorescence emission intensity has also drastically decreased as compared to the other two osmolytes (Fig. 4). These results suggest that proline is most effective in both delaying and suppression of the fibrillation. When the studies were done in the presence of different concentrations of sorbitol (Fig. 3D) it was found that there is only a slight delay in the lag period of insulin fibrillation. In the presence of 50, 100 and 250 mM sorbitol, the lag periods were found to be (447 ± 2), (466 ± 3) and (478 ± 5) min, respectively, with slight decrease in the ThT fluorescence intensity. Figure 4 shows the extent of fibrillation in the presence of the above four osmolytes at 250 mM concentration. Clearly, betaine, citrulline and proline are very effective in preventing the fibrillation of insulin.

Figure 5 (A–D) shows transmission electron microscopic images of insulin taken after 600 min when incubated at 37 °C under stirring condition at 250 rpm, in the presence of 250 mM betaine, citrulline, proline and sorbitol, respectively. It is clear from the that small amorphous aggregates of insulin are formed in the presence of betaine, citrulline and proline (Fig. 5A–C respectively), whereas the presence of sorbitol leads to formation of fibrils having bundle like morphology (Fig. 5D). The TEM images support the results obtained from ThT fluorescence assay that in the presence of betaine, citrulline and proline the fibril formation is inhibited. A slight decrease in the ThT fuorescence intensity in the presence of sorbitol (Fig. 3D) is associated with reduction in the effective area for ThT binding upon bundle formation of fibrils.

### Isothermal titration calorimetry of the interaction of insulin with osmolytes at different stages of fibrillation

To understand the mechanism of inhibition of insulin fibrillation by osmolytes, it is very important to identify the mode of interaction between insulin and the osmolytes at different stages of fibrillation of the protein. As mentioned earlier, although there is substantial literature with regard to protein stabilization by osmolytes^12^,^13^,^14^,^15^,^16^,^17^,^18^,^19^,^20^,^21^,^22^,^23^,^24^,^25^, there is very little data addressing the interaction behavior of inhibitors at different stages of protein fibrillation, especially focusing on the enthalpy of interaction^50^. In this work, isothermal titration calorimetry, an ultrasensitive technique for thermodynamic characterization of intermolecular interactions has been used to address the above issue.

The ITC experiments were performed with native insulin as well as with insulin at different stages of fibrillation with different concentrations of osmolytes. It is comprehended that a protein in the aggregated state may not hold a specific binding site for the inhibitors. However, it is expected that the inhibition will depend on the molecular properties of both the inhibitors and the protein. In all the ITC experiments, the osmolyte solutions were taken in the sample cell and the insulin solution was taken in the syringe. In order to avoid further fibrillation of the protein during the ITC experiments, only 10 injections of insulin were done to determine the enthalpy of interaction [The insulin concentration varied from 3.7 μM to 37 μM during the course of the experiment]. With this arrangement, the ITC experiments could be completed within 50 minutes after equilibration. This procedure allows the insulin taken in the syringe to remain in the desired stage of aggregation during the experiment without getting converted into the next stage of fibrillation.

A typical ITC experiment provides a set of values of the heat liberated or absorbed at different concentrations of insulin in the solution. The limiting standard enthalpy of interaction (ΔH°) is indicative of the nature of solute-solvent interactions without contributions from solute-solute and solvent-solvent interactions. In the cases where the heat of interaction was observed to be concentration dependent, the value of ΔH° was obtained from a linear fit to the experimental data points. If the heat of interaction did not show any concentration dependence, an average of the experimental data points was taken as a limiting standard enthalpy of interaction (The raw ITC data has been included in the supplementary information). Figure 6 shows the value of limiting standard enthalpy of interactions (ΔH°) of insulin with the osmolytes betaine, citrulline, proline and sorbitol at different concentrations and at different stages of fibrillation of insulin.

#### Enthalpies of interaction of insulin with osmolytes at the native stage

Figure 6A shows the ΔH° values for the interaction of native insulin with 50 mM, 100 mM and 250 mM betaine at 25 °C (curve a). It is observed that the interaction is endothermic with ΔH° varying from (6.20 ± 0.30) kJ mol^−1^ to (0.60 ± 0.03) kJ mol^−1^ when the concentration of the osmolyte is changed from 50 to 250 mM. Similar enthalpies of interaction were obtained when insulin was titrated with citrulline, proline and sorbitol. When native insulin is titrated with citrulline (Fig. 6B), proline (Fig. 6C) and sorbitol (Fig. 6D), the ranges of the corresponding enthalpies of interaction are (11.50 ± 0.60) to (7.50 ± 0.40) kJ mol^−1^, (6.80 ± 0.30) to –(0.30 ± 0.02) kJ mol^−1^, and (5.00 ± 0.30) to (2.20 ± 0.11) kJ mol^−1^, respectively at osmoyte concentrations of 50, 100 and 250 mM.

Osmoytes are known to impart conformational stability to proteins when added under denaturation stress conditions^11^,^12^,^13^. The reason for such a stabilization has generally been attributed to preferential hydration of the protein as a result of preferential exclusion of the osmolyte from the protein surface^51^. The observed endothermic enthalpies of interaction of the native insulin with the osmolytes suggest that between the preferential exclusion leading to structuring of water around the protein (expected to be exothermic) and strengthening of the conformation of the protein leading to strengthening of hydrophobic interactions (expected to be endothermic), the latter effect dominates. It is also observed that the enthalpy of interaction has a decreasing trend when the concentration of osmolyte increases. This can be attributed to the reported tendency of the osmolytes to form supramolecular structures at high concentration in aqueous solution, a process which is exothermic in nature^52^.

#### Enthalpies of interaction of insulin with osmolytes at the elongation stage

**Figure 1:**
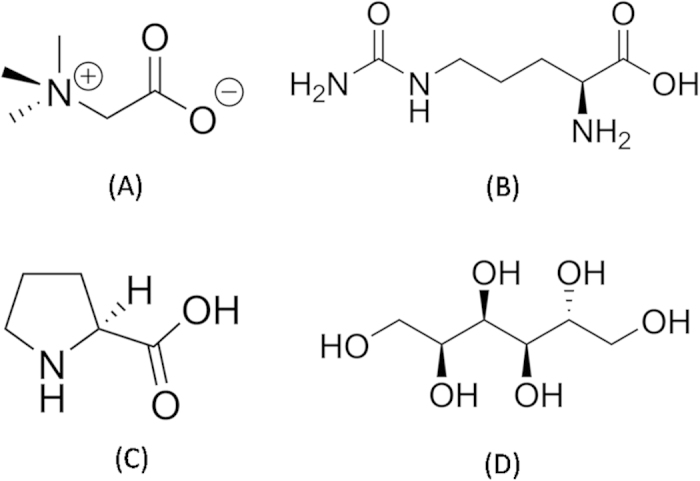
Chemical structures of osmolytes (A) betaine, (B) citrulline, (c) proline and (d) sorbitol.

**Figure 2:**
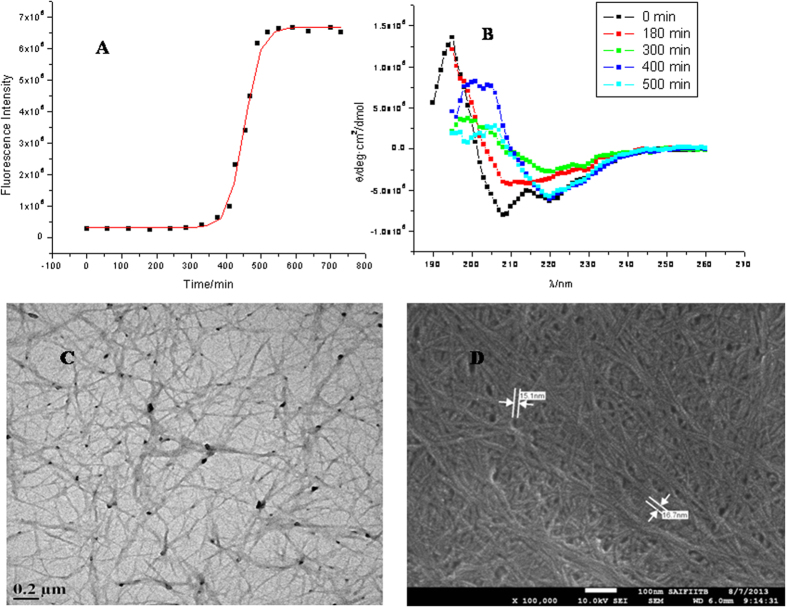
(**A**) Kinetics of the insulin amyloid formation monitored by the binding of ThT with insulin amyloid fibrils, (**B**) far-UV CD spectra of insulin at different time intervals, (**C**) transmission electron microscopic and (**D**) scanning electron microscopic images of insulin fibrils after 600 min of incubation.

The enthalpies of interaction of the insulin fibrils at the elongation phase with the osmolytes are shown in Fig. 6 (curve b). The solution of insulin fibrils taken in the syringe of ITC corresponds to point B of Fig. 2A. A general observation in the figure is that the interaction of insulin with osmolytes at the elongation stage of fibrillation is less endothermic compared to that with the native form. The enthalpy of interaction is also not significantly dependent on the concentration of the osmolyte. The maximum values of the enthalpy of interaction of insulin at this stage with betaine, citrulline, proline and sorbitol are –(0.60 ± 0.03) kJ mol^−1^, –(3.30 ± 0.20) kJ mol^−1^, –(4.20 ± 0.20) kJ mol^−1^ and (1.10 ± 0.05) kJ mol^−1^, respectively. At the elongation stage of the fibrillation process, the protein is expected to be partially unfolded (see Fig. 1B) with greater exposed backbone surface area compared to the native state. Therefore, the prevention of furthering of fibrillation can occur if either the protein is relatively folded back or the osmolyte interacts with the protein and interferes with the association of protein molecules. The extent of reduction in the endothermic enthalpy of interaction of insulin with osmolyte at the elongation stage, though small does suggest two possibilities. The first possibility is its lesser ability to fold the protein back to strengthen intramolecular hydrophobic interactions, and the second possibility is to interact with the exposed polar groups of the protein which can contribute to exothermic heat effects. Relatively small enthalpies of interaction also indicate a possible balance of endothermic and exothermic events. Therefore, it is possible that the osmolytes studied in the present work inhibit the fibrillation both by rendering stabilization to the protein and by interfering in the self assembly process via specific interactions. A close examination of the enthalpy of interaction of insulin at elongation stage with sorbitol suggests absence of polar interactions. The consequence of this effect is reflected in its inability to prevent fibrillation of insulin (see Fig. 5D).

#### Interaction of matured insulin fibrils with osmolytes

The enthalpies of osmolyte interaction with insulin fibrils after maturity are presented in Fig. 6 (curve c). In each case the enthalpy of interaction of the fully grown fibrils is not remarkably different from that with insulin in the elongation stage of the fibrillation process, though the trend is more towards being exothermic. The range of enthalpies of interaction are from –(1.50 ± 0.07) to –(4.00 ± 0.20) kJ mol^−1^ at all the studied concentrations of proline, betaine and citrulline. These results suggest that in each of these three osmolytes the mode of interaction with insulin after the latter has crossed the nucleation stage is nearly the same. The enthalpy of interaction of sorbitol with insulin at matured fibrillation state remains endothermic suggesting absence of significant polar interactions.

The above results suggest that the osmolytes are most effective in preventing fibrillation of insulin if added prior to the onset of the elongation process. This could be attributed to the possibility that the accessible surface of the protein chain for interaction with the osmolytes could be maximum prior to the onset of fibrillation than after the latter has set in.

### Mechanism of prevention of fibrillation of insulin by osmolytes

**Figure 3:**
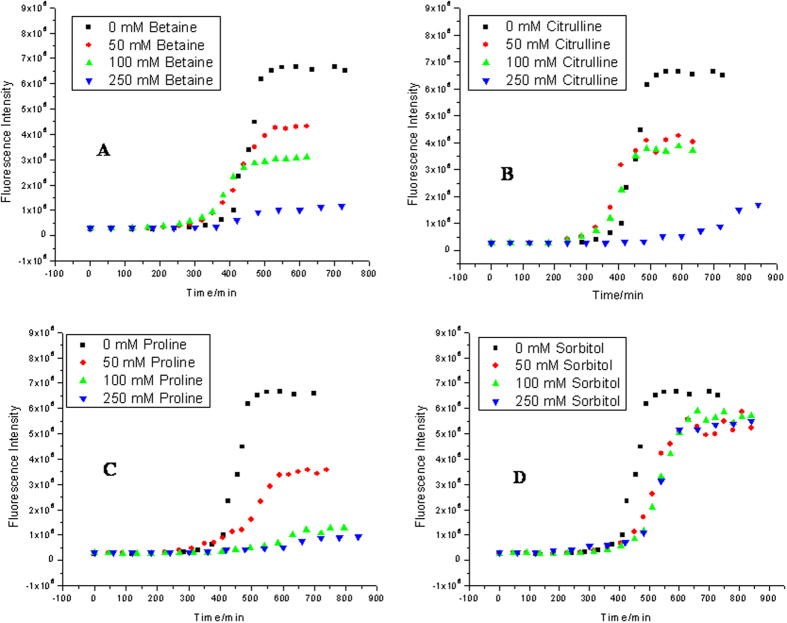
Kinetics of insulin fibril extension in absence and in presence of different concentration of osmolytes (A) betaine, (B) citrulline, (C) proline and (D) sorbitol studied by monitoring the changes in fluorescence emission intensity as a function of time.

Amongst betaine, citrulline, proline and sorbitol, the first three osmolytes are seen to effectively inhibit the fibrillation of insulin (Fig. 3). Betaine and citrulline have significantly reduced the extent of fibrillation. On the other hand, proline has not only suppressed but also delayed the fibril formation. ITC results suggest the combined effect of preferential exclusion andspecific interactions by these three osmolytes, leading to inhibition of fibrillation. Looking at the structures of these osmolytes (Fig. 1), it appears that the interactions would be largely electrostatic in nature, although proline can also provide a hydrophobic face from its side chain. The fact that proline is most effective in inhibiting fibrillation seems to suggest that it interferes with the stacking of β-sheets which is, generally, largely driven by hydrophobic interactions. Figure 7 schematically summarizes the model for fibrillation inhibition by the three osmolytes. In the native conditions (in the absence of osmolytes), there is a specific structure of water on the outer surface of the protein. Partial unfolding leads to greater exposure of the protein surface which leads to self-association and fibrillation. In the presence of the osmolytes, two processes can happen: (i) enhanced hydration of the protein surface, strengthening of intra-molecular hydrophobic association, which imparts compactness to the protein, which in turn inhibits inter-molecular hydrophobic association and (ii) direct interaction of osmolytes with the protein surface. Both these factors inhibit fibrillation of the protein. The relative contributions of these two factors will depend upon the structural and chemical characteristics of the individual osmolytes. Certain degree of amorphous aggregation of the partially folded molecules, however, is still possible.

**Figure 4:**
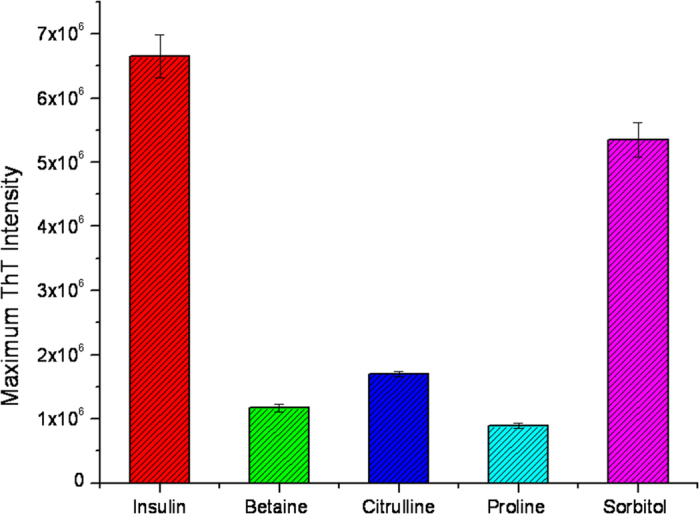
Maximum ThT intensity observed during insulin fibril formation in presence of 250 mM osmolytes.

**Figure 5:**
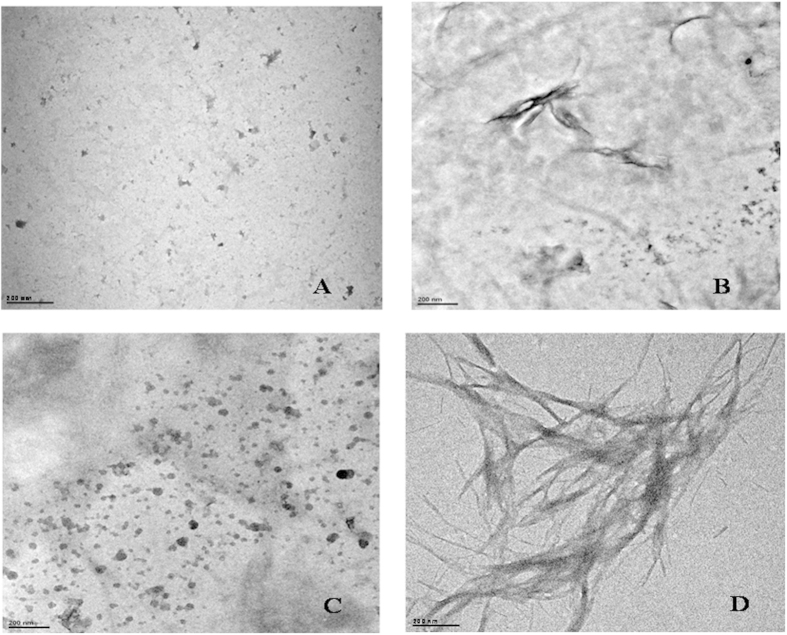
Transmission electron microscopic images of insulin after 600 min of incubation in presence of 250 mM (A) betaine, (B) citruine, (C) proline and (D) sorbitol.

**Figure 6:**
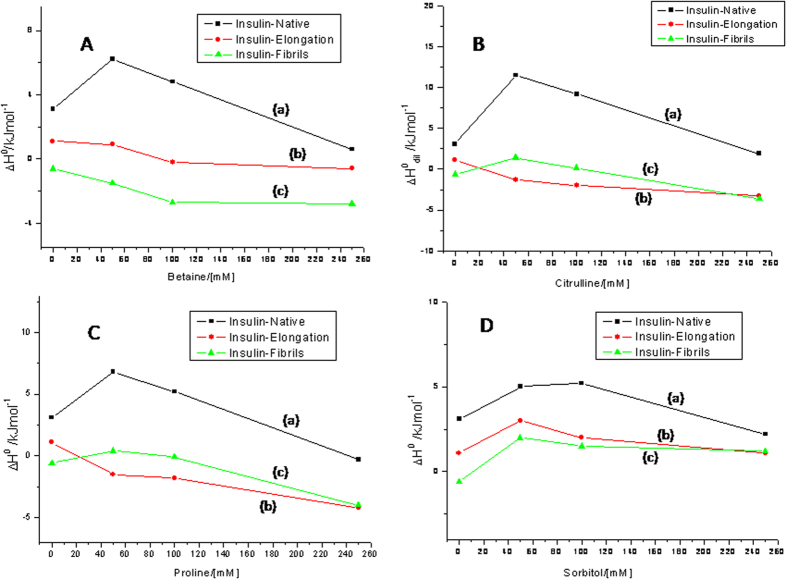
Limiting enthalpies of interaction of insulin with (A) betaine, (B) citrulline, (C) proline, and (D) sorbitol at different stages of fibrillation.

**Figure 7:**
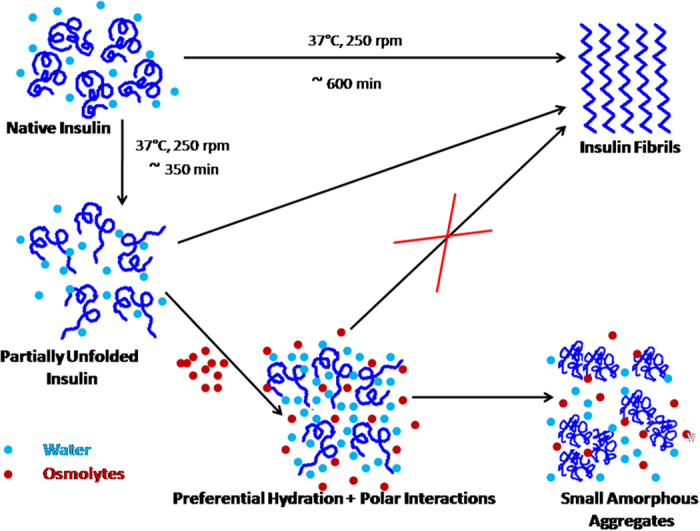
Schematic representation of mechanism of inhibition of insulin fibrillation by osmolytes.

In contrast, sorbitol is less effective in inhibiting fibrillation which is also reflected in ThT fluorescence intensity (Figs 3D and 4). Isothermal titration calorimetric results for sorbitol also show different behavior than with the other three osmolytes. In general, the enthalpy of interaction of native insulin with sorbitol is seen to be less endothermic than with the other osmolytes. The enthalpy of interaction of sorbitol with insulin at elongation and matured fibrillation state also remains endothermic suggesting absence of significant polar interactions which are clearly seen in the values of enthalpies of interaction with the other osmolytes. These results suggest that sorbitol, in spite of having several polar hydroxyl groups, is unable to engage in polar interactions with the insulin chain that could prevent self-association of the chain. Rather, its mechanism of action is primarily, ‘preferential exclusion from the surface’ and its overall efficacy of fibrillation inhibition is less than that of the other osmolytes.

## Conclusions

The present study demonstrates that osmolytes betaine, citrulline, proline and sorbitol inhibit fibrillation of insulin, and also provides valuable insights into the mechanism of their action. A variety of biophysical tools including ThT fluorescence, CD, TEM and ITC were used to derive information on the process of fibrillation and also on the thermodynamics of osmolyte interactions with insulin at different stages of fibrillation. Out of these four studied osmolytes, proline was found to be most effective in delaying the onset, as well as in inhibition of fibrillation. Betaine and citrulline have very little effect on the lag period but the extent of fibrillation is significantly reduced. Transmission electron microscopic images in the presence of betaine and proline show dispersed amorphous aggregates, whereas a mixture of amorphous aggregates with few fibrils was seen in the presence of citrulline. On the other hand clusters of fibrils were visible in the presence of sorbitol. From ITC measurements, we conclude that the interaction of native insulin with the osmolytes endorse strengthening of intramolecular hydrophobic interactions in the protein as a result of preferential exclusion of the osmolyte leading to greater compaction. We also conclude that polar as well as non-polar interactions with the protein, prior to the onset of fibrillation, play crucial roles in the prevention of inter-molecular association and thus of fibrillation. The results also suggest that the osmolytes are most effective in preventing fibrillation of insulin, if added prior to the onset of elongation stage.

## Materials and Methods

### Chemicals and buffers

Bovine pancreatic insulin (>0.95), L-proline (>0.99), citrulline (>0.95), trehalose (>0.95), sorbitol (>0.95), glycine betaine (>0.95) and thioflavin T (dye content 0.65–0.75) were procured from Sigma-Aldrich Chemical Company USA. The listed purities of these compounds, on mass fraction basis, are given in the parenthesis. All the solutions were prepared in milliQ water from Merk Millipore. The experiments were performed in 20 mM phosphate buffer at pH 2.0. The stock solution of insulin was prepared initially in the phosphate buffer (20 mM, pH 2.0) and dialyzed overnight at 4° C against the buffer with at least three changes of the latter. All the other solutions were prepared in the final dialysate buffer.

### *In vitro* insulin amyloid fibrillation

The concentration of the protein was determined on a UV-1800 Shimadzu UV-visible spectrophotometer, using an extinction coefficient A_1_ _cm_^1%^ = 1.0 at 276 nm^53^. The samples of Insulin at a concentration of 3.0 mg/ml were incubated at 37 °C under stirring condition at 250 rpm to induce fibrillation.

### Fluorescence spectroscopy

The steady state fluorescence measurements were done on a Fluoromax-4 spectrofluorometer (Horiba Scientific) with excitation and emission slit widths fixed at 5 nm. A stock solution of ThT was prepared in phosphate buffer (20 mM, pH 2.0). The concentration of ThT was determined by using an extinction coefficient *E* = 26,620 M^1^ cm^−1^ at 412 nm^54^. The ThT molecules were selectively excited at λ_ex_ = 450 nm^47^ and emission was monitored at λ_em_ = 482 nm^47^. At different time intervals an aliquot of incubated insulin solution was mixed with ThT solution such that the final concentrations of insulin and ThT for the fluorescence measurements were 5 μM and 50 μM, respectively. The acquired data from ThT fluorescence measurements were fitted to the sigmoid curve depicted by the following equation^55^


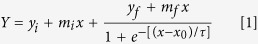


Here *Y* is the fluorescence intensity, *x* is time, and *x*_0_ is the time to reach 50% of maximal fluorescence. Thus, the apparent rate constant, *k*_app_, for the growth of fibrils is given by 1/*τ*, and the lag time is given by *x*_0_ – 2*τ*.

### Circular dichroism spectroscopy

The far-UV CD spectra of insulin was analysed to monitor the changes in the secondary structure of insulin during fibrillation. The CD spectra were recorded on a JASCO-810 spectropolarimeter by taking aliquots at different time intervals from the incubated insulin sample. The concentration of protein and path length used was 5 μM and 0.2 cm, respectively, for the far-UV CD experiments. The spectropolarimeter was thoroughly purged with nitrogen gas before starting the experiments. Each spectrum was baseline corrected and was taken as an average of three accumulations at a scan rate of 50 nm min^−1^, and a response time of 1 s. The molar ellipticity was calculated from the observed ellipticity *θ* as


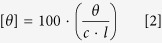


where *c* is the concentration of the protein in mol dm^−3^and *l* is the path length of the cell in centimeters.

### Transmission Electron Microscopy

In order to visualize insulin amyloid fibrillation the images were acquired on a JEOL JEM-100B Transmission Electron Microscope which operates at an accelerating voltage of 80 kV. For this purpose an aliquot of 10 μl from the incubated solution was placed on the Formvar-coated 300 mesh copper grids. After a few minutes of drying, the sample containing copper grids were negative stained with 2% aqueous uranyl acetate solution. After pre-rinsing with large volumes of water, a 0.22 μm filter was used to filter the stains. Uranyl acetate is known to produce high electron density, image contrast, and impart fine grained impression to the image^56^.

### Isothermal Titration Calorimetry

The interaction of insulin amyloid fibrils with osmolytes was studied by using ultra sensitive isothermal titration calorimeter (VP ITC form Microcal LLC). The fibril solution was titrated into the sample cell containing buffer or appropriate amount of the osmolyte in aliquots using a rotating stirrer-syringe of 250 μl capacity. The reference cell was filled with the respective buffer. The experiments were designed for a total of 10 consecutive injections, each having a volume of 10 μl of 0.517 mM native insulin solution or heat induced fibril solution into buffer or osmolyte solution in the cell. The duration between consecutive injections was 10 s with an interval of 4 min between each injection. The control experiments were done to measure heats of dilution of the osmolytes and protein under same experimental conditions. After dilution corrections, the ITC profiles were analyzed to determine the heat of interaction by using Origin 7.0 software supplied by Microcal LLC.

## Additional Information

**How to cite this article**: Choudhary, S. *et al.* Inhibition of insulin fibrillation by osmolytes: Mechanistic Insights. *Sci. Rep.* **5**, 17599; doi: 10.1038/srep17599 (2015).

## Supplementary Material

Supplementary Information
