# Supplementary material for: Inhibition of insulin fibrillation by osmolytes: Mechanistic Insights
Source: Sci Rep. 2015 Nov 30;5:17599. doi: 10.1038/srep17599 (PMC4663473; doi:10.1038/srep17599)
Supplement: Supplementary Information [file srep17599-s1.pdf]

# Inhibition of insulin fibrillation by osmolytes: Mechanistic Insights

**Sinjan Choudhary[1], Nand Kishore[2] and Ramakrishna V. Hosur[1,3]**

[1]UM-DAE Centre for Excellence in Basic Sciences, Mumbai University campus, Mumbai 400098, India.Contact No. 022-26524984

[2]Department of Chemistry, Indian Institute of Technology-Bombay, Mumbai 400076, India.

[3]Department of Chemical Sciences, Tata Institute of Fundamental Research, Homi Bhabha Road, Mumbai 400005, India, hosur@tifr.res.in, Contact No. 022-22782488

**Supplementary Information:**

**Figure legends:**

**Figure S1:** Representative ITC profiles for the titration of insulin with buffer at 37 °C after incubation of 0 min [■], 420 min [●], and 600 min [▲].

**Figure S2:** Reprentative ITC profile showing heat of interaction of insulin at different stages of fibrillization in with 50 mM (A) betaine, (B) citrulline, (C) proline, and (D) sorbitol at 25 °C and pH 2.0.

**Figure S3:** Reprentative ITC profile showing heat of interaction of insulin at different stages of fibrillization in with 100 mM (A) betaine, (B) citrulline, (C) proline, and (D) sorbitol at 25 °C and pH 2.0.

**Figures:**

**Figure S1**

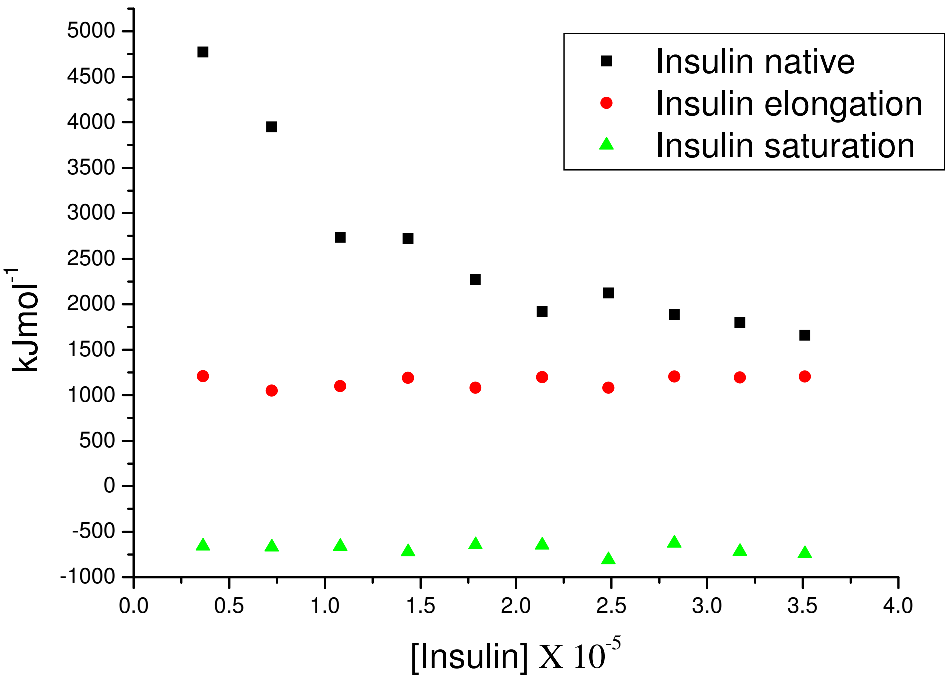

**Figure S2**

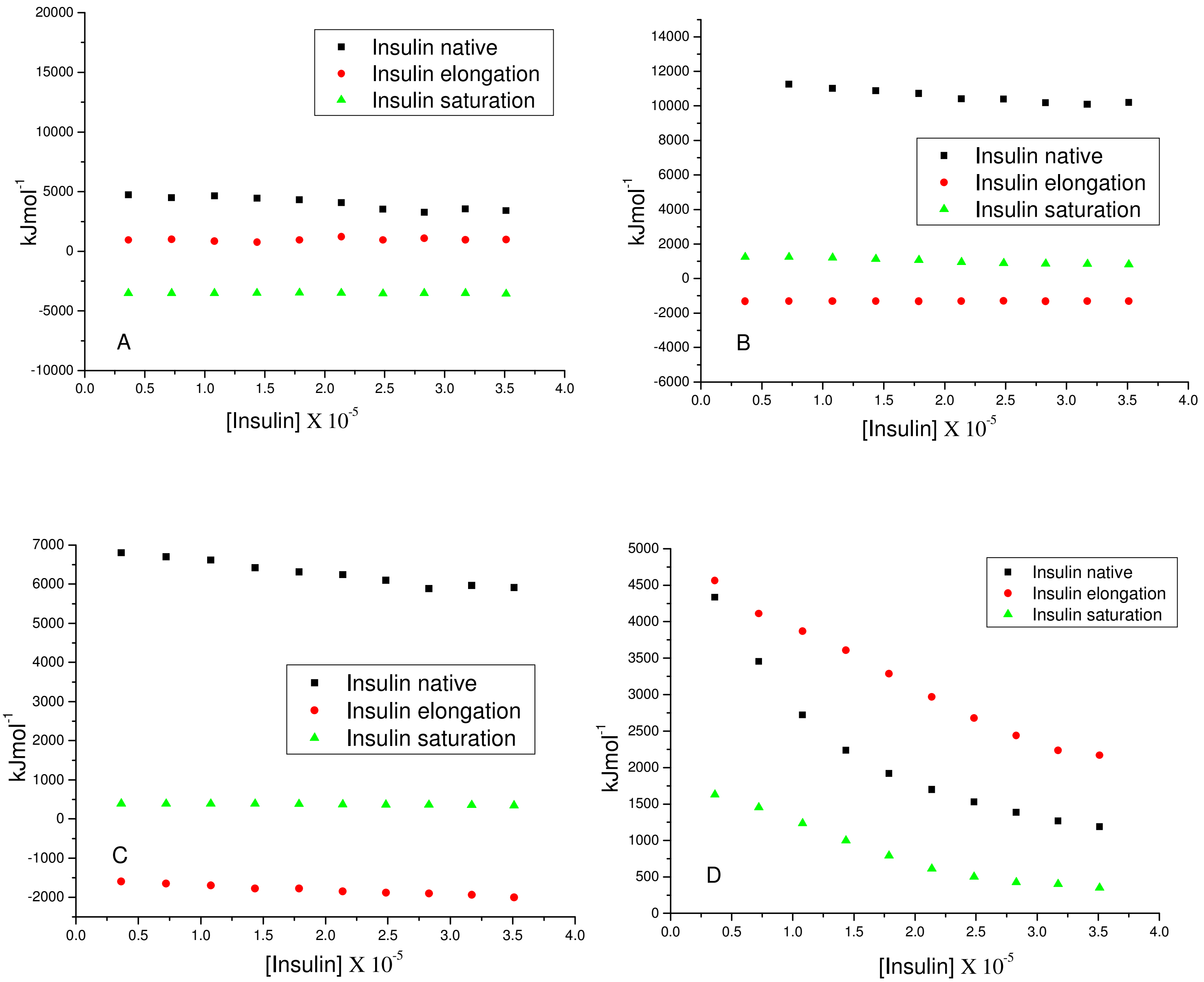

Insulin native
Insulin elongation
Insulin saturation
kJmol-1
[Insulin] X 10-5
A
B
C
D

**Figure S3**

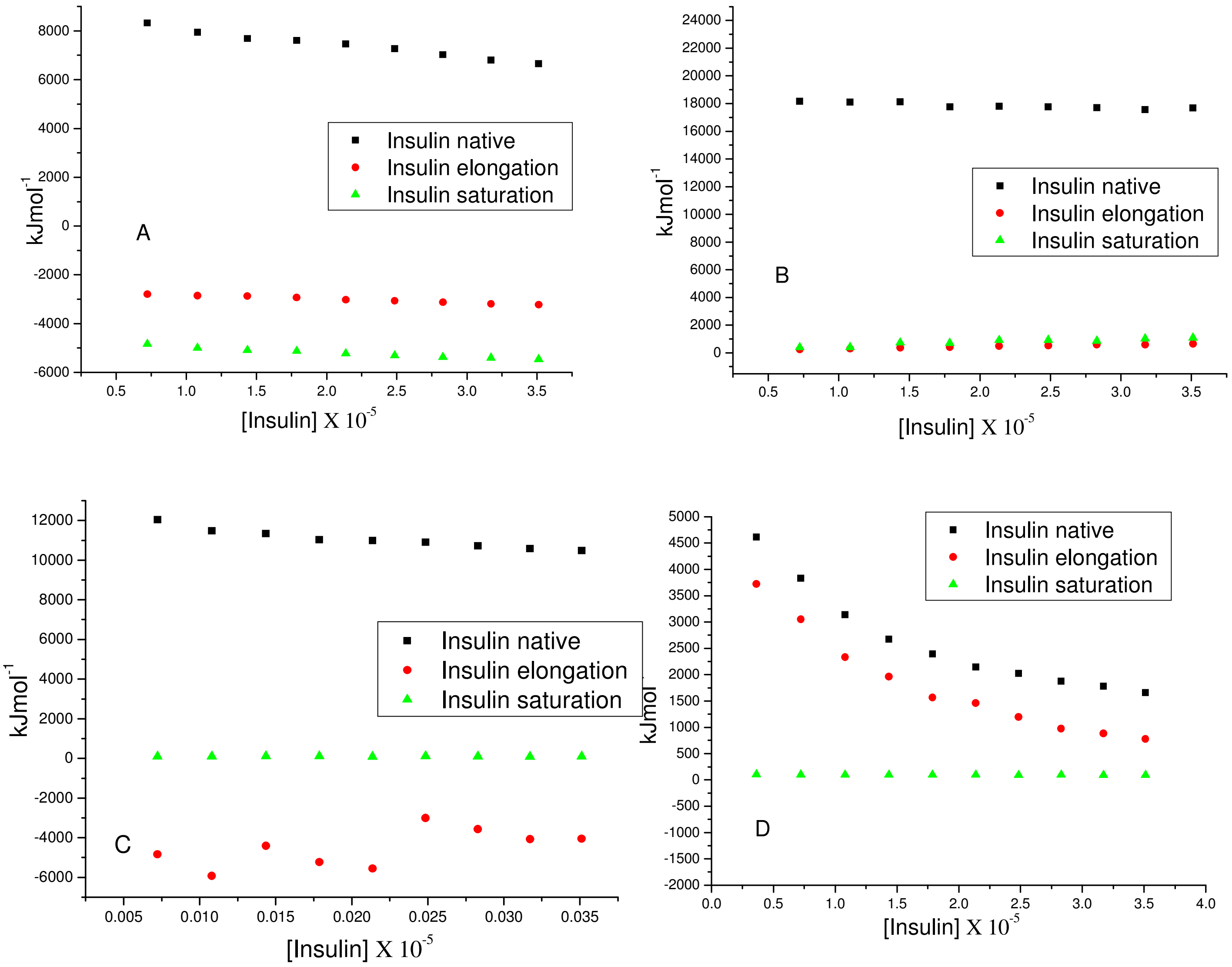

kJmol-1
A
Insulin native
Insulin elongation
Insulin saturation
[Insulin] X 10-5
B
C
D
